# Cost to conduct screening, brief intervention, and referral to treatment (SBIRT) in healthcare settings

**DOI:** 10.1186/1940-0640-8-S1-A7

**Published:** 2013-09-04

**Authors:** Carolina Barbosa, Alexander J Cowell, William N Dowd, Justin Landwehr, Jeremy W Bray

**Affiliations:** 1 Behavioral Health and Criminal Justice Research Division, RTI International, Chicago, IL, USA; 2 Behavioral Health and Criminal Justice Research Division, RTI International, Research Triangle Park, NC, USA; 3 Behavioral Health and Criminal Justice Research Division, RTI International, Rockville, MD, USA

## Introduction

This paper presents data from the Substance Abuse and Mental Health Services Administration (SAMHSA) cross-site evaluation of the third cohort of SAMHSA grantees implementing screening, brief intervention, and referral to treatment (SBIRT) services in selected sites. Cost estimates for SBIRT service provision are needed because in the present environment, healthcare expenditures will need to be justified fiscally. The objective of the current paper is to determine the costs of delivering individual SBIRT components as well as the program as a whole.

## Methods

A multifaceted data collection protocol was employed to collect cost data from grantee agencies and selected sites of the third cohort of Substance Abuse and Mental Health Services Administration’s (SAMHSA) SBIRT program. First, detailed labor and space utilization data were collected through direct observation of SBIRT practitioners in their duties over complete shifts. Next, administrators at both grantee and site levels were asked to complete questionnaires that collected data on wages, administrative labor, and non-labor costs. Finally, selected practitioners were asked about irregular and infrequent activities over the past month that would have been missed during direct observation.

## Results

Using the detailed labor utilization data coupled with wage data collected from administrators, precise estimates of the cost to deliver each component of SBIRT will be presented. Additionally, program-level costs will be presented using data from the administrator questionnaires and practitioner past-month experiences.

## Conclusions

These results provide insight into the cost of hosting an SBIRT program in a healthcare setting. Further research is needed to compare the costs with available funding streams for SBIRT services.

